# Influence of Workload on Primary Care Nurses’ Health and Burnout, Patients’ Safety, and Quality of Care: Integrative Review

**DOI:** 10.3390/healthcare8010012

**Published:** 2020-01-03

**Authors:** Darío Hilario Pérez-Francisco, Gonzalo Duarte-Clíments, José María del Rosario-Melián, Juan Gómez-Salgado, Macarena Romero-Martín, María Begoña Sánchez-Gómez

**Affiliations:** 1 General Emergency Service, La Palma Health Area, 38700 Santa Cruz de La Palma, Spain; dariohperez01@hotmail.com; 2 School of Nursing, Candelaria N.S. University Hospital, University of La Laguna, 38010 Santa Cruz de Tenerife, Spain; extgduartcl@ull.edu.es (G.D.-C.); begonasanchez@gmail.com (M.B.S.-G.); 3 Department of Family and Community Nursing, Multiprofessional Teaching Unit of Family and Community Assistance, 38010 Santa Cruz de Tenerife, Spain; serjan9501@hotmail.com; 4 Department of Sociology, Social Work and Public Health, University School of Social Work, University of Huelva, 21071 Huelva, Spain; 5 Safety and Health Posgrade Program, University Espíritu Santo, 091650 Guayaquil, Ecuador; 6 Centro Universitario de Enfermería Cruz Roja, University of Sevilla, 41009 Sevilla, Spain; mromero@cruzroja.es

**Keywords:** health care quality, nursing, patient safety, primary health care, professional burnout, workload

## Abstract

The increase in the demand for care has not led to a proportional growth in the number of primary care nurses. This imbalance is related to the decrease in the quality of care and patient safety, and also to the impact on the health of the professional group. The objective of this study is to identify relationships between overload, illness of the nurse, professional exhaustion, quality and safety in the services; and differentiate study methods and instruments for measuring the phenomena. For this, a comprehensive and structured review of the literature following the scoping review model is performed. The studies on which this review is based allow us to recognize that the scope of this phenomenon is global. The review includes 45 studies that show that there is a high pressure of care for Primary Care nursing, who suffer many alterations of their health due to burnout syndrome and that this situation contributes to the impairment of the quality of care and patient safety. However, for future lines, new evidence is needed to determine the degree of relationship between the high pressure suffered by Primary Care nursing and the attainment of health goals for professionals and patients.

## 1. Introduction

In the different healthcare models or systems that exist in the world, the term “efficiency” stands out as a pillar that guarantees their sustainability. Therefore, the best use of available resources is a central part of the work managers are faced with. Achieving health objectives with the lowest possible expenditure in times of crisis [1] has pushed management engineering to the limit. One of the sectors concerned is health staff [2], especially after cuts in human resources and materials [3].

Within healthcare staff, nursing is a very large group with the capacity to impact on reducing costs for a system that has also suffered the effects of the current economic situation [4]. As symptoms, lines of research [5,6,7,8] have appeared to try and determine how this affects the recipient of assistance: the patient. These works have typically focused on problems that affect hospital care, leaving aside those that affect the gateway to healthcare: primary care. In recent years, the scientific production in this line regarding primary care has grown. It has tried to investigate how it affects the recipient of the assistance. There have also been authors who have followed a parallel and less known path [9]: trying to know what happens with health workers and how this is related with the outcome of care.

The clearest consequence of the cuts in nursing is the increase of the workload [10,11,12,13], while it is still a hardly quantifiable variable [14,15]. Within the profession, there are mechanisms that help soften the effects of this situation [16,17,18,19,20]. However, professional exhaustion [21] appears, whereby primary care variables related to overload, such as the deterioration of the staff’s health status [22,23,24,25], decreased the quality of care [26], and a negative effect on the patient’s safety [27,28] emerged. This has been declared of great importance in the national health system [29] and the nursing profession [30]. The definition of work overload varies depending on the study and different criteria and tools are used, so it is a matter that depends on the individual perception of the researchers in each case. Some of the criteria used have been a lower ratio of nurses [6,8], subjective perception of overload [12,26,30], or several questionnaires [6,8,10,11,12,23].

Regarding the deterioration of nurses’ health, burnout and professional exhaustion [31] have become more important, as well as their relationship with job satisfaction [32,33,34], quality of life at work [35], and suicidal risk [36], in addition to quality of care and patient safety and satisfaction [37]. The scope of the problem is global, as there are studies in different labour contexts at the international level [38,39,40,41,42]. Although protective factors [43,44] have been identified, one of the decisive issues in the emergence of this phenomenon evidenced in numerous studies is the increase in the pressure on healthcare [45]. This fact shows a growing trend in the future due to the profile of the patient who demands assistance [15]. Therefore, it is reasonable to think that if the care workload increases and, with it, burnout, this will further affect related phenomena such as staff’s health status, the quality of care, and patients’ safety.

There are studies that established a relationship between increased workload and increased mortality in patients in hospital care [8] and, in addition to this increase in mortality, they also demonstrated a direct relationship between the increased number of patients assigned to a nurse and the increase in staff who suffer burnout [6]. Although there are studies on hospital care, it is complex to identify studies in primary care that quantify the relationship between workload and the health of the professional, the quality of care, and patient safety. In the same way, it is difficult to find studies that implement improvement measures for the aforementioned aspects and that determine their repercussions. In addition, a gold standard is not available for the method and the measuring instruments of this relationship. This research gap is especially important in the area of primary care, given that most of the available studies are focused on the hospital setting. Why is it important for primary care? Health services have primary care as a gateway, and this has been suffering a decrease in the quality of the service provided with less resources [10], a high working pace [12], and insecurity in patients [22] in recent years. The effects of professional burnout [37] and work overload [26,27] are behind this phenomenon. Regarding the quality of care, the main aspects affected are the increase in costs and the decrease in productivity of professionals [4], the increase in hospital stays [4,7,8], demoralized [11] and sick [17,24,25,32] personnel, outdated knowledge [25], lower quality of services provided [16] and worsening of records [26]. Regarding patient safety, an increase of the adverse effects derived from patient care [5,6,7,16], mortality [4,5,6,7], and preventable errors [27] are observed.

Therefore, the objective of this review is to identify relationships between overload, nurses’ diseases, burnout, quality, and safety during the services. This paper also aims to identify methods of study, instruments of measurement of the phenomena, and strategies of improvement.

## 2. Materials and Methods

To meet the described objectives, a structured review of the scientific literature was carried out following the method of scoping review described by Arksey and O’Malley [46] with a particular emphasis on articles published in Spanish or Portuguese. This method has a narrative and descriptive characteristics to obtain an overview and a synthesis of the available studies. The steps followed were research question, identifying relevant studies, study selection, charting data, collating, summarising, and reporting the results. This review methodology is particularly useful to examine a specific issue, systematically map and integrate the literature, and identify key concepts, theories, evidence available or, on the contrary, research gaps.

A search strategy that follows the PICO format was established. This question was made visible in the descriptors in health science (DeCS, for its Spanish acronym) and the medical subject headings (MeSH). A search of articles was conducted in the following databases: Biblioteca Virtual de Salud (BVS), Base de Datos de la Fundación Index sobre Cuidados de Salud en Iberoamérica (CUIDEN), National Library of Medicine (MEDLINE), Joanna Briggs Institute (JBI), collection of databases of controlled clinical trials in health sciences (COCHRANE), and more. The following keywords were used for the search strategy: primary health care, nursing, workload, burnout, occupational health, patient safety, quality of care. They were combined using the booleans operators AND and OR for electronic searches conducted in the mentioned databases, as is shown in Tables S1 and S2.

Additionally, an electronic search was completed with a reference search on the literature quoted in the articles selected.

**Figure 1:**
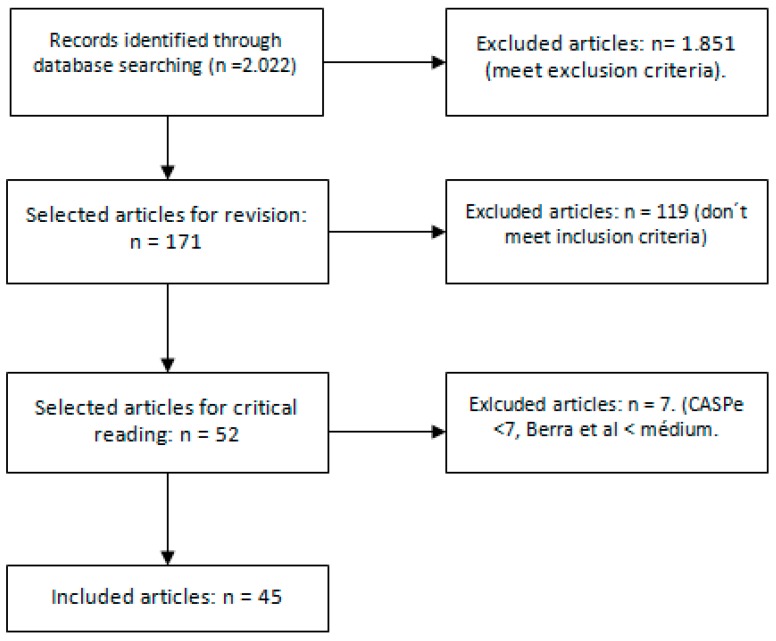
Search strategy flowchart.

The study selection process is summarized in Figure 1. Initially, 2022 references were identified and after the screening process, 45 studies were selected for this review.

### 2.1. Search Limits

The inclusion criteria are:

- Articles published in the last 10 years.
- In English, Spanish, or Portuguese.
- Full-text available at the time of search.
- Primary care related works that correspond to the search topic.
- Hospital care works that correspond to the subject of search: only reviews or clinical trials, prominent authors, and large samples.
- Assistance to healthy and non-healthy adults.

The exclusion criteria are:

- Studies on physicians’ burnout where nurses are not included.
- Works in which the methodology is not described, the subjects of the study are not clear, or the inclusion criteria in the case of the reviews are not described.

### 2.2. Structure and Analysis

The selected articles are structured according to the objectives, on the one hand, towards overload, burnout, and quality-safety of the services and the relations that may exist between them and, on the other hand, towards the methods of study, measurement instruments of the phenomena, possible interventions, and expected results.

The selected studies are blindly peer-reviewed following the CASPe instruments by selecting those with a score higher than or equal to 7, and the checklist by Berra et al. (2008) [47] for descriptive studies; in this case, those that reach a medium or high level are selected. Discrepancies are resolved through discussion and consensus among the research team. Evidence synthesis is performed by the JBI classification [48]. 

A total of 45 studies were included in this review and, according to the method followed, these were 12 secondary studies (11 reviews and 1 meta-analysis) and 33 primary studies (1 randomised controlled trial, 1 quasi-experimental study, 1 case-control, 21 descriptive, 7 qualitative, 1 quanti-qualitative, and 1 expert opinion). Regarding the critical appraisal, out of the articles assessed using the CASPe tool, 8.7% scored 7; 39.1% scored 8; 43.5% scored 9; and 8.7% scored 10. Descriptive studies were assessed using the Berra et al. tool; 47.6% obtained medium level of quality and 52.4% obtained high level of quality. Regarding grades of recommendation, 84.8% studies obtained grade B and 15.2% studies obtained grade A.

### 2.3. Assessment Tools

The assessment tools used in the reviewed studies are summarised in Table 1.

## 3. Results

The main objective of this review is to identify the relationships between overload, nurse illness, burnout, quality, and safety during services. We identified articles that prove the existence of a relationship between work overload, illness and burnout of nurses, and the quality and safety of the service. The relationship between emotional exhaustion, quality of care and patient safety is demonstrated by the highest level of evidence in Salyers’ meta-analysis [37].

In this recently published meta-analysis [37], the relationship between burnout, patient quality and patient safety is established. A higher staff burnout proportion has an impact on the quality of care, which implies a significantly negative relationship between burnout and perceived quality, as can be seen in several studies. In the same way, some studies included in this meta-analysis establish a negative relationship between burnout and the level of safety perceived by the patient, with the finding that this relationship is stronger in the nursing collective than in the physicians’ one and is greater in Europe than in America. All the above-described is in line with these previously described relationships, which do nothing but confirm what was stated.

There are articles that relate overload, burnout, quality, and patient safety. There are overlaps that identify the problem of nursing overload, effects on health/ burnout, decreased quality of care, and effects on patient safety [6,16]. Increased workload has effects on nurses, manifested through burnout [6]. In the light of nursing presenteeism [16], which is increasing due to the lack of replacement, job insecurity, etc., there is an increase in the probability of patients suffering adverse events due to poor performance per hour worked. These result in decreased safety and quality in the care the patient receives.

As for the articles that relate overload, burnout, and quality [22,36,42], they coincide in talking about nursing overload, effects on health/burnout, and decreased quality of care. In line with the previously mentioned point, work overload decreases patient safety, and in this case, an effect on the safety of the professional [22] (accidents at work) is added, as well as mental disorders [36,49] derived from high pressure. A relationship [42] between professional exhaustion and a lower-quality performance is established for many specific tasks that directly affect the patient, such as low-quality prescription, as well as a relationship between burnout and worse quality indicators such as less case resolution at the healthcare level.

Examining the articles that establish a relationship between overload, safety, and quality [2,8,26,30], it is verified that an increased healthcare pressure implies having less time for the direct attention to the patient, and likewise, less time for the recording of the medical history [26], with the associated damage to the continuity of the processes. The mentioned increase in healthcare pressure implies a decrease in the attitude of safety towards the patient [30] and in the practices that affect the medical staff [8]. The upward imbalance of workload for not respecting the nurse-patient ratio established is manifested in problems such as increased infections in patients [2].

This document also aims to identify study methods, instruments for measuring phenomena and improvement strategies that complement the main outcomes of the study. The results obtained are structured in these three different and interconnected themes:

1. Methods of study.
2. Instruments used in the studies.
3. Interventions that improve burnout, patient safety, and quality of care.
4. Relationship between workload and burnout, patient safety and quality.

### 3.1. Methods of Studys

It is important to emphasize the following when dealing with reviews and quasi-experimental studies: the management of nursing consultations is improved by carrying out interventions such as group EfH (Education for Health) [19], telenursing [18], or mindfulness training [44]. These interventions, aimed at increasing the efficiency of time allocated to care, use of resources, reducing health problems and work-related accidents [22] in staff and incidents affecting the user, are not purely of an assistance nature. Work overload is related [17,32] with increased burnout [31], a negative effect on health, the deterioration of job satisfaction, and with the presence of errors [27] that affect the patient. When the demand is purely of an assistance nature, increased [5] nurse-patient ratio significantly decreases the adverse effects in the latter, in addition to being economically beneficial for the institutions [4]. Nursing is a profession associated to presenteeism [16], a term opposed to absenteeism and that helps to better channel the broad existing healthcare demand.

In the RCT [9], it is stated that nursing uses an important amount of time for the assistance activity on demand, in addition to the programmed activity in the consultation. Users’ satisfaction with the resolution of their problems is high.

In the descriptive studies, we emphasize the following: high job satisfaction [11] in nurses improves the user’s satisfaction [33]. Work overload, which in turn is related to decreased quality of care, has an influence on this satisfaction [10], as well as the errors made in user’s care and safety of the patient, and the burnout, for which different prevalence are identified [21,36,38,39,41,42,43,45].

Qualitative studies follow the line of the above said and identify primary care nursing day-to-day risks, including healthcare overload [12,13,35]. The inadequacy of the available human resources is not the only conditioning factor for primary care nursing, but also [3] inadequate material and environmental resources that affect life quality at work. The data records [26] are deficient regarding high-pressure healthcare situations.

### 3.2. Instruments Used in the Studies

The most used instrument for burnout is the Maslach Burnout Inventory (MBI). The prevalence of burnout is variable depending on the study (Table 2). However, all of them agree that this phenomenon is common in primary care nursing and in the different contexts in which it is studied. Even though there are significant differences in the percentages of burnout found, it must be considered that the criteria according to the score obtained are different in each case, and that also certain studies [42,45] consider that there are cases in which only one dimension is affected.

A cut-off point [50] to determine the presence or lack of existence of burnout if the Maslach Burnout Inventory is used is not found. A single score is not found either, but 3 different subscales are rated: emotional exhaustion (EE), personal accomplishment (PA), and depersonalization (D). Low, medium, or high levels of burnout are determined according to the scores for each dimension as follows: low EE ≤ 18, PA ≥ 40, and D ≤ 5; medium EE 19–26, PA 34–39, and D 6–9; high EE ≥ 27, RP ≤ 33, and D ≥ 10. These scores correspond to the MBI-HSS51 version (MBI human services survey) used for healthcare staff. As it is said above, these cut-off points are not those generally adopted in the works studied.

Semi-structured interview. When analysing this, we note that the items explored are records quality, occupational risks, quality of life/conditions at work, interpersonal relationships, and socio-demographic variables. The studies in which they are used are heterogeneous and are reduced to qualitative studies.

### 3.3. Interventions That Improve Burnout, Patient Safety, and Quality of Care

Burnout in nursing decreases with interventions like the improvement of work organization [21,28] in relation to the pace of work, and a better organization of work shifts considering the staff preferences. In addition, the improvement [31] of salaries and an increased support on the part of the organization influences job satisfaction. Mindfulness techniques [42] performance, greater teamwork, and an improvement of the relationship with managers [43] appear as further necessary interventions to reduce the prevalence of burnout.

Patient safety improves [6,8,9,23] with an increased nurse-patient ratio, which implies fewer errors, less mortality, and longer nursing care [7,24,25], and facilitates the application of nursing processes and the realization of higher quality caring plans. A higher level of nursing staff education [30] is also attributed to a lower level of errors in patient care.

The quality of care improves with telenursing [17], considering the various ways of providing the service such as through telephone calls—proactive and/or reactive, videoconferencing, or push-button devices. Less readmissions, better therapeutic control, improvements in diseases and well-being, and fewer in-person consultations [17], are recorded. On the other hand, the decrease of the workload facilitates recording of the patient’s medical history [32], also facilitating the transmission of information without errors and omissions between professionals. Nurses [20] have a high sense of helping others (engagement) which make it easier for them to overcome the obstacles they encounter in the performance of their daily work. Lastly [14], a standardized language within the profession is needed to improve communication regarding the most common interventions in primary care.

### 3.4. Relationship between Workload and Burnout, Patient Safety and Quality

The results are shown in Table 3.

## 4. Discussion

The articles included in this review give proof of the existence of a relationship between the exposed variables. Work overload that primary care nurses suffer and the associated burnout they may experience as a result of their type of work affect the nurses themselves by worsening their health status. However, this will also affect patients, as their safety is not guaranteed, as there is an increase in nursing mistakes related to overload and burnout, as well as the consequent mortality and, as stated in this review, the quality of care patients receive is also hampered by these factors. All these effects have economic implications for the system [17,22,31,32]. The studies that support this review allow to know the global scope of this connection [10,11,12,13,17,21,27,31,35,36,38,39,41,42,43,45,49], that cannot be attributed to the existing particularities of a specific field.

In addition to the relationships already indicated, the evidence shows that there are interventions whose application, which is presented in a simple way, can help to improve the experience of nurses regarding their provision of care, and indirectly, the experience of patients under their care. Thus, organisational measures such as adapting work shifts and the functioning of services to staff preferences [21,28], diversifying care with initiatives such as telecare [17], empowering stress-related capacities [42], decreasing the nurse-patient ratio [6,8,9,23], and supporting and improving communication with the organisation [31] appear as possible solutions to the phenomena we dealt with in this review. As already present, the high sense of assistance (commitment) that nurses have is shown as an improvement in organizational terms [20].

Articles have been identified that prove the existence of a relationship between nurses’ work overload, illness and burnout, and the quality and safety of the service they provide. However, measuring burnout involves talking about the most commonly used instrument, the Maslach BurnOut Inventory (MBI). Although this instrument confers the possibility of comparing results, the review carried out shows an important limitation. This limitation consists of the great variability of criteria for considering emotional exhaustion, depersonalisation and low performance at work. We observe non-standardised cut-off points or points that are independently established for each dimension. In fact, burnout cut-off points differ in all the found studies. This element of variability in interpreting is known by the scholars, and allows adapting the conclusions found in the studies to the reality of the organisation [50].

Despite this difficulty, in the selected studies we can see a relationship between the workload in primary care nursing and its effects on the health of nurses, the decrease of the quality of care, and the errors made in the same context. The studies in which this review is supported make it possible to recognize that the scope of this phenomenon is global [10,11,12,13,17,21,27,31,35,36,38,39,41,42,43,45,49].

Another difficulty found is that the methods of approach are heterogeneous, as well as the measuring instruments [2,6,8,12,13,16,22,26,27,30,31,35,36,37,42]. For example, the nine studies that deal with patient safety find that the instruments are limited to accounting for the proportion of errors per cases attended; the questionnaires in descriptive studies are heterogeneous and, from the qualitative point of view, the semi-qualitative Delphi technique is only used in one study. In the rest, the interview technique is used. In the same way, the interventions on these problems and their results are heterogeneous.

Although the heterogeneity found is important, it should be noted that the effects of work overload in primary care nursing should be addressed to limit the deficits: errors in care services, increased mortality, decreased quality of care, nurses’ disease, and burnout. All these effects carry important economic implications [17,22,31,32].

Regarding the recommendations made to improve the burnout we find: the start-up of telenursing [17], the improvement of work organization [21,28], a better organization of the work shifts [21,28], the improvement of salaries, a greater support from the organisation [31], an increase in the nurse-patient ratio [6,8,9,23], and the performance of mindfulness techniques [42] as well as physical activity [51] and healthy aging strategies [52]. The high sense of help (engagement) nurses have is shown as an improvement in the organisation [20].

### Limitations

In addition to the limitations already pointed out, such as those referring to the Maslach questionnaire or the adequacy of the interventions, another limitation of this study is the low number of studies in the field of primary care, as the knowledge base on this topic is traditionally related to the field of hospital care. This limitation is minimized by choosing current evidence and presenting an adequate application of the methodology, a fact that has been confirmed after the critical reading of all the articles. Despite limiting the search to articles in Spanish, English, and Portuguese, evidence has been found in very diverse media.

Although, most of the articles included in this review were published in Portuguese and Spanish, it includes researches conducted in a wide international scope: Brazil [3], the United States of America [7], the United Kingdom and Canada [17], Spain [18], Chile [20], South Africa [26], Switzerland, Australia and New Zealand [27], Germany [33], Thailand and China [34], Portugal [40], Argentina, Uruguay, Mexico, Colombia, Guatemala, El Salvador, Ecuador and Peru [41].

## 5. Conclusions

The impact of nurses’ working conditions on patients’ care is a major concern for the health system. The reviewed articles identified a relationship between the variables included in this paper. Nurses’ workload was related to burnout and decreased safety and quality of care. Workload and pressure at work limited the time that nurses could dedicate to patients care and also their attitude towards patients’ safety is more relaxed. Workload was also related to a negative effect on nurses’ health, deterioration of job satisfaction, and the concurrence of errors and adverse effects.

The reviewed articles suggested interventions in order to reduce burnout such as improvements in the work organisation, support on the part of the organisation, improvements in the relationship with managers, team-working, and mindfulness techniques. Regarding patients’ safety, an increased nurse-patient ratio was suggested, that could lead to fewer errors, less mortality, longer nursing care, and higher quality caring plans. The quality of care could be improved through tele-nursing, as well as improving the quality of medical records and the use of a nursing standardised language to improve communication.

The evidence summarised in this review could help in implementing organisational interventions to improve nurses’ working environments and, consequently, quality of care and patients’ satisfaction.

## Figures and Tables

**Table 1:** Tools used in the studies.

	Tool	Reference ^a^
**Nursing Overload.**	Qualitative interview.	[12,26,35]
	Specific questionnaires of each study	[6,8,10,11,23]
	EIPST, EACT	[12]
	No tool used	[1,2,7]
**Burnout/Effects on Nursing Health.**	Maslach	[38,39,40,41,42,43,44,45,47]
	WHQOL-100	25
	Specific questionnaires of each study	[6,10,24,36,39], [40] (3 questionnaires), [44] (3 questionnaires), [45]
	No tool used	[1]
**Patient Safety.**	Qualitative interview	[12]
	Qualitative interview	[12,13]
	No tool used	[2]
**Quality of Care.**	Delphi technique	[28]
	Specific questionnaires of each study	[6,8,30]
	Qualitative interview	[3]
	EUROPEP	[33]
	SAQ	[30]
	Specific questionnaires of each study	[6,8]
	No tool used	[1,2]

^a^ The numbering corresponds with the citation in the text.

**Table 2:** Use of MBI in studies.

Study	%Burnout	Cut-off Points	Sample	Score *	Burnout *
[31] Gómez-Urquiza JL, Monsalve-Reyes CS et al. 2017	Various	-	-	Important part presents high EE, high D and low P	-
[32] Khamisa N et al. 2013.	Various	-	-	Important part presents high EE, high D and low P	-
[36] Tomás-Sábado J et al. 2010	Not stated	EE > 24 D > 9 PA > 39	N = 146	High EE: 23.9% Hig D: 13% Low PA: 9.6%	High EE High D Low PA
[38] Palmeira Sarmento Silva SC et al. 2015.	6.7–10.8%	EE > 25 D > 8 PA < 3 4	N = 198	High EE: 43% High D: 17% Low PA: 32%	High EE High D Low PA
[39] Domínguez Fernández JM, Herrera Clavero F, et al. 2012	17.2%	EE > 26 D > 9 PA < 34	N = 200	High EE: 26% High D: 34% Low PA: 71%	High EE High D Low PA
[40] Rui Gomes A et al. 2009.	15% EE	Does not specify	N = 286	High EE: 15% High D: 4% Low PA: 1%	Does not specify
[41] Grau A et al. 2009	11% (Global)/14% Spain	Does not specify	N = 11.530	Gives means.	High EE High D Low PA
[42] de Dios del Valle R, Franco Vidal A. 2007	36.6%	EE > 31 D > 13 PA < 30	N = 145	High EE: 20% High D: 11% Low PA: 57.9%	One of the three dimensions with high degree
[43] Vilà Falgueras M et al. 2015	17.2%	EE > 28 D > 9 PA < 34	N = 879	High EE: 38.2% High D: 23.8% Low PA: 7.7%	High EE High D or High EE High PA or High D Low PA
[45] Navarro-González D et al. 2015	39.3% 22.4%	EE > 27 D > 10 PA < 34	N = 78	High EE 15.7% High D: 18.5% Low PA: 47.2%	One of the three dimensions with high degree
[49] Cañadas-De La Fuente, G.A., et al. 2016	Between 42.5% and 44.1%	-	N = 1021	High EE High D High PA	High EE High D High PA

* EE: emotional exhaustion, D: depersonlisation, PA: personal accomplishment.

**Table 3:** Relationship between workload and burnout, patient safety and quality.

Reference	Workload	Burnout	Patient Safety	Quality of Care
[4] M. Dall, T et al.	If it decreases: (more ratio of nurses)	Does not specify	Increases	Increases
[5] L. Kane, R et al.	If it decreases: (more ratio of nurses)	Does not specify	Increases	Does not specify
[6] H Aiken, L et al.	If it increases: (less nurse ratio)	It appears	Decreases	Decreases
[7] Needleman, J et al.	If it decreases: (more time per patient)	Does not specify	Decreases	Increases
[8] H Aiken, L et al.	If it increases: (high pressure/ high work rate)	Does not specify	Decreases	Does not specify
[10] Scherlowski Leal David, HM et al.	If it increases: (high pressure/high work rate)	It appears	Does not specify	Does not specify
[11] Pérez-Ciordia, I et al.	If it increases:	It appears	Does not specify	Decreases: demoralized professional
[16] Reyes Revuelta, JF et al.	If it increases:	Does not specify	Decreases	Decreases
[17] Joyce, K et al.	If it increases:	Does not specify	Does not specify	Decreases: sick professional
[18] González-Esteban, MP et al.	If it decreases: telenursing	Does not specify	Increases	Increases
[19] Echevarría-Zamanillo, MM et al.	If it decreases: (more time per patient)	Does not specify	Does not specify	Increases: patient satisfaction
[21] da Silveira Maissiar, D et al.	If it increases:	Does not specify	Does not specify	Decreases: exhausted professional
[24] Elaine Tomasi, E et al.	If it increases:	Does not specify	Does not specify	Decreases: sick professional
[25] Silva Fernandes, J et al.	If it increases:	Does not specify	Does not specify	Decreases: sick profesional/not updated knowledge
[26] Shihundla, RC et al.	If it increases:	Does not specify	Does not specify	Decreases: worse records
[28] Martínez Ques, AA et al.	If it increases:	Does not specify	Does not specify	Decreases
[29] Agra Varela, Y et al.	If they are not organized	Does not specify	Decreases	Does not specify
[30] Fernanda Paese, G et al.	If it increases:	Does not specify	Decreases	Does not specify
[32] Khamisa, N et al.	If it increases: stress related to care pressure	It appears	Does not specify	Decreases: sick professional
[37] Salyers, MP et al.	If there is a high workload	It appears	Does not specify	Decreases
[38] Palmeira Sarmento Silva, SC et al.	If there is a high workload	It appears	Does not specify	Does not specify
[39] Domínguez Fernández, JM et al.	If there is a high workload	It appears	Does not specify	Does not specify
[40] Rui Gomes, A et al.	If more hours are worked	It appears	Does not specify	Does not specify
[41] Grau, A et al.	If guards are made	It appears	Does not specify	Decreases
[42] de Dios del Valle, R et al.	If there is a high workload	It appears	Does not specify	Decreases
[45] Navarro-González, D et al.	If there is a high workload	It appears	Does not specify	Does not specify

## Supplementary Materials

The following are available online at https://www.mdpi.com/2227-9032/8/1/12/s1, Table S1: PRISMA 2009 Checklist- Search strategy in databases. Table S2: PRISMA 2009 Checklist- Manual search strategy.

## References

[1] Karanikolos M., Mladovsky P., Cylus J., Thomson S., Basu S., Stuckler S., Mackenbach J.P., McKee M. (2013). Financial crisis, austerity, and health in Europe. Lancet.

[2] Montgomery K.L. (2003). Health care at the crossroads, strategies for addressing the evolving nursing crisis. Nurs. Educ. Perspect..

[3] Corrêa Daubermann D., Pamplona Tonete V.L. (2012). Qualidade de vida no trabalho do efermeiro da Atenção Básica à Saúde. Acta Paulista Enfermagem.

[4] Dall T.M., Chen Y.J., Seifert R.F., Maddox P.J., Hogan P.F. (2009). The Economic Value of Professional Nursing. Med. Care.

[5] Kane R.L., Shamliyan T.A., Mueller C., Duval S., Wilt T.J. (2007). The Association of Registered Nurse Staffing Levels and Patient Outcomes. Med. Care.

[6] Aiken L.H., Clarke S.P., Sloane D.M., Sochalski J., Silber J.H. (2002). Hospital nurse staffing and patient mortality, nurse burnout and job dissatisfaction. JAMA.

[7] Needleman J., Buerhaus P., Mattke S., Stewart M., Zelevinsky K. (2002). Nurse-staffing levels and the quality of care in hospitals. N. Engl. J. Med..

[8] Aiken L.H., Sloane D.M., Bruyneel L., Van den Heede K., Griffiths P., Busse R., Diomidous M., Kinnunen J., Kózka M., Lesaffre E. (2014). Nurse staffing and education and hospital mortality in nine European countries: A retrospective observational study. Lancet.

[9] Kinnersley P., Anderson E., Parry K., Clemen J., Archard L., Turton P. (2000). Randomised controlled trial of nurse practitioner versus general practitioner care for patients requesting “same day” consultations in primary care. BMJ.

[10] Scherlowski Leal David H.M., Chaves Mauro M.Y., Gomes Silva V., de Souza Pinheiro M.A., Henriques da Silva F. (2009). Organização do trabalho de enfermagem na atenção básica: Uma questão para a saúde do trabalhador. Texto Contexto Enfermagem.

[11] Pérez-Ciordia I., Guillén-Grima F., Brugos A., Aguinaga Ontoso I. (2013). Satisfacción laboral y factores de mejora en profesionales de atención primaria. Anales Sistema Sanitario Navarra.

[12] Cesar-Vaz M.R., Soares J.F.S., Figueiredo P.P., Azambuja E.P., Sant’Anna C.F., Costa V.Z. (2009). Percepción del riesgo en el trabajo en salud. Revista Latino-Americana Enfermagem.

[13] Batista Gil Nunes M., do Carmo Cruz Robazzi M.L., de Souza Terra F., Chaves Mauro M.Y., Gollner Zeitoune R.C., de Oliveira Secco I.A. (2010). Riscos ocupacionais dos enfermeiros atuantes na atenção à saúde da famílía. Revista Enfermagem UERJ.

[14] Bonfim D., Rapone-Gaidzinski R., Monique Santos F., de Souza Gonçales C., Togeiro Fugulin F.M. (2012). Identificação das intervenções de enfermagem na Atenção Primária à Saúde: Parâmetro para o dimensionamento de trabalhadores. Revista Escola Enfermagem USP.

[15] Gené Badia J., Borràs Santos A., Contel Segura J.C., Camprubí Casellas M.D., Cegri Lombardo F., Heras Tebar A. (2011). Nursing workload predictors in Catalonia (Spain): A home care cohort study. Gaceta Sanitaria.

[16] Reyes Revuelta J.F. (2014). Presentismo en Enfermería. Implicaciones en seguridad del paciente. Posibilidades de control y reducción. Enfermería Global.

[17] Joyce K., Pabayo R., Critchley J.A., Bambra C. (2010). Flexibilización laboral y sus efectos sobre la salud y el bienestar de los empleados. Biblioteca Cochrane Plus.

[18] González-Esteban M.P., Ballesteros-Álvaro A.M., Crespo-de las Heras M.I., Pérez-Alonso J. (2015). Teleenfermería: Nuevo Enfoque de Intervenciones Eficaces en Atención Primaria.

[19] Echevarría-Zamanillo M.M., Fraile-Caviedes C., Diez-Sánchez T.J., Rodríguez-Ferrer C., Gamarra-Llousa M., Chicote-Aylagas P.N. (2009). Organizamos de Forma Adecuada el Tiempo en Las Consultas de Enfermería en Atención Primaria. https://www.saludcastillayleon.es/investigacion/es/banco-evidencias-cuidados.

[20] Fernández Merino C., Yañez Gallardo R. (2014). Describiendo el engagement en profesionales de enfermería de atención primaria de salud. Ciencia Enfermería.

[21] da Silveira Maissiar D., Lauter L., Del Pai D., Petri Tavares J. (2015). Contexto de trabalho, prazer e sofrimento na atenção básica em saúde. Revista Gaúcha Enfermagem.

[22] Guedes dos Santos J.L., Vierira M., Cardoso Assuitii L.F., Gomes D., Schlindwein Meirelles B.H., de Azevedo dos Santos S.M. (2012). Risco e vulnerabilidades nas práticas nos proffisionais de saúde. Revista Gaúcha Enfermagem.

[23] Elaine Tomasi E., Augusto Facchini L., Xavier Piccini R., Elaine Thumé E., Silva da Silveira D., Vinholes Siqueira F. (2008). Perfil sócio-demográfico e epidemiológico dos trabalhadores da atenção básica à saúde nas regiões Sul e Nordeste do Brasil. Cadernos Saúde Pública.

[24] Elaine Tomasi E., Castro Sant’Anna G., Oppelt A.M., Magalhães Petrini R., Vianna Pereira I., Tomasi Sassi B. (2007). Condições de trabalho e automedicação em profissionais da rede básica de saúde da zona urbana de Pelotas, RS. Revista Brasileira Epidemiologia.

[25] Silva Fernandes J., de Souza Castro Miranzi S., Hemiko Iwamoto H., dos Santos Tavares D.M., dos Santos C.B. (2012). Relação dos aspectos profissionais na qualidade de vida dos enfermeiros das equipes Saúde da Família. Revista Escola Enfermagem USP.

[26] Shihundla R.C., Lebese R.T., Maputle M.S. (2016). Effects of increased nurses’ workload on quality documentation of patient information at selected Primary Health Care facilities in Vhembe District, Limpopo Province. Curationis.

[27] Makeham M., Dovey S., Runciman W., Larizgoitia I. (2008). Methods and Measures Used in Primary Care Patient Safety Research.

[28] Martínez Ques A.A., Hueso Montoro C., Gálvez González M. (2010). Fortalezas y amenazas en torno a la seguridad del paciente según la opinión de los profesionales de enfermería. Revista Latino-Americana Enfermagem.

[29] Agra Varela Y., Casado Durández P., Palanca Sánchez I., García Díaz M.J., Álvarez González C., Castrodez Sanz J.J. (2015). Estrategia de Seguridad del Paciente del Sistema Nacional de Salud. Ministerio de Sanidad.

[30] Fernanda Paese G., Marcon Dal Sasso T. (2013). Cultura da segurança do paciente na atenção primária à saúde. Texto Contexto Enfermagem.

[31] Gómez-Urquiza J.L., Monsalve-Reyes C.S., San Luis-Costas C., Fernández-Castillo R., Aguayo-Estremera R., Cañadas-de la Fuente G.A. (2017). Factores de riesgo y niveles de burnout en enfermeras de atención primaria: Una revisión sistemática. Atención Primaria.

[32] Khamisa N., Peltzer K., Oldenburg B. (2013). Burnout in Relation to Specific Contributing Factors and Health Outcomes among Nurses: A Systematic Review. Int. J. Environ. Res. Public Health.

[33] Szecsenyi J., Goetz K., Campbell S., Broge B., Reuschenbach B., Wensing M. (2011). Is the job satisfaction of primary careteam members associated with patient satisfaction?. BMJ Qual. Saf..

[34] Liu Y., Aungsuroch Y., Yunibhand J. (2016). Job satisfaction in nursing: A concept analysis study. Int. Nurs. Rev..

[35] Schrader G., Palagi S., Silveira Padilha M.A., Tuerlinckx Noguez P., Buss Thofehrn M., Dal Pai D. (2012). Trabalho na Unidade Básica de Saúde: Implicações para a qualidade de vida dos enfermeiros. Revista Brasileira Enfermagem.

[36] Tomás-Sábado J., Maynegre-Santaulària M., Pérez-Bartolomé M., Alsina-Rodríguez M., Quinta-Barbero R., Sergi Granell-Navas S. (2010). Síndrome de burnout y riesgo suicida en enfermeras de atención primaria. Enfermería Clínica.

[37] Salyers M.P., Bonfils K.A., Luther L., Firmin R.L., White D.A., Adams E.L., Rollins A.L. (2017). The Relationship between Professional Burnout and Quality and Safety in Healthcare: A Meta-Analysis. J. Gen. Intern. Med..

[38] Palmeira Sarmento Silva S.C., Prado Nunes M.A., Rocha Santana V., Prado Reis F., Machado Neto J., Oliveira Lima S. (2015). A síndrome de burnout em profissionais da Rede de Atenção Primária à Saúde de Aracaju, Brasil. Ciência Saúde Coletiva.

[39] Domínguez Fernández J.M., Herrera Clavero F., Villaverde Gutiérrez M.C., Padilla Segura I., Martínez Bagur M.L., Domínguez Fernández J. (2012). Síndrome de desgaste profesional en trabajadores de atención a la salud en el área sanitaria de Ceuta. Atención Primaria.

[40] Rui Gomes A., Fernando Cruz J., Cabanelas S. (2009). Estresse Ocupacional em Profissionais de Saúde: Um Estudo com Enfermeiros Portugueses. Psicologia Teoria Pesquisa.

[41] Grau A., Flichtentrei D., Suñer R., Prats M., Braga F. (2009). Influencia de factores personales, profesionales y transnacionales en el síndrome de burnout en personal. Sanitario hispanoamericano y español. Revista Española Salud Pública.

[42] de Dios del Valle R., Franco Vidal A. (2007). Prevalencia de burnout entre los profesionales de Atención Primaria, factores asociados y relación con la incapacidad temporal y la calidad de la prescripción. SEMERGEN.

[43] Vilà Falgueras M., Cruzate Muñoz C., Orfila Pernas F., Creixell Sureda J., González López M.P., Davins Miralles J. (2015). Burnout y trabajo en equipo en los profesionales de Atención Primaria. Atención Primaria.

[44] Martín Asuero A., Rodríguez Blanco T., Pujol-Ribera E., Berenguera A., Moix Queraltó J. (2013). Evaluación de la efectividad de un programa de mindfulness en profesionales de atención primaria. Gaceta Sanitaria.

[45] Navarro-González D., Ayechu-Díaz A., Huarte-Labiao I. (2015). Prevalencia del síndrome de burnout y factores asociados a dicho síndrome en los profesionales sanitarios de Atención Primaria. SEMERGEN.

[46] Arksey H., O’Malley L. (2005). Scoping studies: Towards a methodological framework. Int. J. Soc. Res. Methodol..

[47] Berra S., Elorza-Ricart J.P., Estrada M.D., Sánchez E. (2008). Instrumento para la lectura crítica y la evaluación de estudios epidemiológicos transversales. Gaceta Sanitaria.

[48] The Joanna Briggs Institute (JBI) (2013). The Joanna Briggs Institute New Levels of Evidence.

[49] Cañadas-De la Fuente G.A., Albendín-García L., de la Fuente E.I., San Luis C., Gómez-Urquiza J.L., Cañadas G.R. (2016). Síndrome de burnout en profesionales de enfermería que realizan jornada física complementaria en servicios de cuidados críticos y urgencias. Revista Española Salud Pública.

[50] González Correales R., De la Gándara Martín J.J. (2004). El Médico Con Burnout.

[51] López Bueno R., Casajús Mallén J.A., Garatachea Vallejo N. (2018). La actividad física como herramienta para reducir el absentismo laboral debido a enfermedad en trabajadores sedentarios: una revisión sistemática. Rev. Esp. Salud. Pública.

[52] García Candil M.T., Lecuona Irigoyen A., Iknurov Mollov A., Navincopa Quezada A.M., García López V. (2019). Abordaje preventivo del envejecimiento saludable por los servicios de prevención de riesgos laborales. Rev. Esp. Salud. Pública.

