# Supplementary material for: Influence of Workload on Primary Care Nurses’ Health and Burnout, Patients’ Safety, and Quality of Care: Integrative Review
Source: Healthcare (Basel). 2020 Jan 3;8(1):12. doi: 10.3390/healthcare8010012 (PMC7151231; doi:10.3390/healthcare8010012)
Supplement: Supplementary file 1 [file healthcare-08-00012-s001.pdf]

Table S1 Search strategy in databases.

Databases	DeCS / MeSH	Date of search	Articles found	Articles selected for revision	Articles selected for critical reading	Excluded articles	Valid articles
BVS (SciElo, LiLACS)	TOTAL		**842**	65	24	4	20
	"enfermería de atención primaria" AND "carga de trabajo"	14/11/2016	6	4	2	0	2
	"enfermería de atención primaria" AND "agotamiento profesional"	18/11/2016	9	2	0	0	0
	"enfermería de atención primaria" AND "seguridad del paciente"	28/11/2016	2	0	0	0	0
	"enfermería de atención primaria" AND "Calidad de la atención de salud"	07/12/2016	21	6	1	0	1
	"enfermería de atención primaria" AND "salud laboral"	10/12/2016	3	1	0	0	0
	"nursing" AND "workload" AND "occupational health"	14/12/2016	406	23	10	1	9
	"nursing" AND "workload" AND "patient safety"	23/12/2016	345	22	8	2	6
	"Primary health care" AND workload AND "quality of health care"	26/12/206	50	7	3	1	2
PubMed	TOTAL		**779**	68	19	1	18
	"primary care nursing" AND workload	03/01/2017	15	11	6	0	6
	"Primary health care" AND Burn out professional"	04/01/2017	7	4	2	0	2
	"Primary health care" AND workload AND "Quality of health care"	05/01/2017	51	9	1	0	1
	"Primary health care" AND workload AND "Occupational health"	07/01/2017	14	2	0	0	0
	"Primary health care" AND "workload" AND "burnout, professional"	09/01/2017	25	6	2	0	2
	"nursing" AND "workload" AND "Occupational health"	10/01/2017	370	19	5	1	4
	"nursing" AND "workload" AND "Patient safety"	18/01/2017	297	17	3	0	3
TripDatabase	TOTAL:		**144**	1	0	0	0
	"nursing" AND "workload" AND "Occupational health"	25/01/2017	25	0	0	0	0
	"Primary health care" AND "burnout, professional"	28/01/2017	7	1	0	0	0
	"nursing" AND "workload" AND "Patient safety"	30/01/2017	96	0	0	0	0
	"Primary health care" AND workload AND "Quality of health care"	02/02/2017	8	0	0	0	0
	"Primary health care" AND workload AND "Occupational health"	03/02/2017	3	0	0	0	0
MEDES	TOTAL		**90**	5	1	0	1

	"enfermería de atención primaria" AND "salud laboral"	05/02/2017	1	1	0	0	0
	"atención primaria en salud" AND "seguridad del paciente"	20/02/2017	69	4	1	0	1
CUIDEN	TOTAL		**11**	8	0	0	0
	"enfermería de atención primaria" AND "salud laboral"	26/02/2017	2	2	0	0	0
	"atención primaria en salud" AND "calidad de la atención de salud"	27/02/2017	1	1	0	0	0
	"Atenção primária à saúde" AND "carga de trabalho"	28/02/2017	3	2	0	0	0
	"Atenção primária à saúde" AND "saúde do trabalhador"	01/03/2017	5	3	0	0	0
Cochrane / C. plus	TOTAL		**78**	13	1	0	1
	"atención primaria de salud" AND enfer* OR "calidad de la atención de salud"	09/03/2017	9	4	1	0	1
	"atención primaria de salud" AND enfer* OR "seguridad del paciente"	12/03/2017	28	5	0	0	0
	"atención primaria de salud" AND enfer* OR "salud laboral"	15/03/2017	19	4	0	0	0

Table S2. Manual search strategy

Database	Search date	Articles found	Articles selected for revision	Articles selected for critical reading	Excluded articles	Valid articles
Banco de Evidencias en Cuidados (SACyL) (Caring Evidence Bank)	30/03/2017	**65**	4	2	0	2
Reference search	26/04/2017	**13**	7	5	2	3
Investen - Joana Briggs	01/05/2017	0				
Agencia de evaluación de tecnologías sanitarias (Health Technologies Assessment Agency)	01/05/2017	0				
INAHTA	02/05/2017	0				
TESEO (doctoral theses in Spain)	05/05/2017	0				
National Institute for Health and Care Excellence (NIEE)	05/05/2017	0				
**TOTAL**		2022	171	52	7	45
